# Incidence of bifid pancreatic duct in pancreaticoduodenectomy and its impact on clinically relevant postoperative pancreatic fistula

**DOI:** 10.3389/fonc.2022.934978

**Published:** 2022-08-17

**Authors:** Liu Ouyang, Hao Hu, Gang Nie, Li-xue Yang, Zhi-ping Huang, Chen-ming Ni, Zhuo Shao, Kai-lian Zheng, Wei Jing, Bin Song, Gang Li, Xian-gui Hu, Gang Jin

**Affiliations:** ^1^ Department of the Hepatobiliary and Pancreatic (HBP) Surgery, Changhai Hospital, Naval Medical University, Shanghai, China; ^2^ Department of Biliary Tract Surgery II, Eastern Hepatobiliary Surgery Hospital, Naval Medical University, Shanghai, China; ^3^ Department of Hepatobiliary Surgery, General Hospital of Southern Theatre Command, Guangzhou, China

**Keywords:** bifid pancreatic duct, morbidity, pancreaticoduodenectomy, CR-POPF, nomogram

## Abstract

**Objectives:**

This study aimed to examine the incidence of bifid pancreatic duct (BPD) in pancreaticoduodenectomy (PD) and clarify its impact on clinically relevant postoperative pancreatic fistula (CR-POPF).

**Background:**

Until now, all the literature about BPD during PD are published as case reports, and the incidence of BPD in PD and its impact on CR-POPF remain unknown.

**Results:**

A total of 438 consecutive PDs were divided into two groups: the former year group and the latter year group. The former year group included 215 consecutive PDs, while the latter year group included 223. In the latter year group, we found 16 BPDs during PD (O-BPD); the incidence of O-BPD is 7.17%. Of them, there were eight patients who had BPD in the preoperative imaging (I-BPD). All the I-BPDs are O-BPDs; which means that 50% of O-BPDs were a single pancreatic duct in the preoperative imaging (I-SPD). There were 17 I-BPDs in the 438 consecutive PDs; the incidence of I-BPD is 3.88%. In the former year group, the rate of severe complications of I-BPD and I-SPD is 77.78% and 27.18%, respectively (*p* = 0.003); the rate of CR-POPF of I-BPD is higher than I-SPD, 55.56% vs. 27.18%, but there were no statistically significant differences. In the latter year group, the rate of severe complications of O-BPD and O-SPD is 50% and 18.36%, and the rate of CR-POPF of O-BPD and O-SPD is 37.5% and 22.22%, respectively; both of them have statistically significant differences, and the *p*-value is 0.003 and 0.006, respectively. In the subgroup analysis, both the rate of severe complications and the rate of CR-POPF of I-BPD were higher than O-BPD, 77.78% vs. 50%, and 55.56% vs. 37.5%, but there were no statistically significant differences in both of them; the *p*-value is 0.174 and 0.434, respectively. Univariate and multivariate analyses showed that BPD was an independent risk factor of CR-POPF.

**Conclusions:**

The incidence of O-BPD in PD is 7.17%, 50% of O-BPDs were I-SPD, and the incidence of I-BPD is 3.88%. BPD is an independent risk factor of CR-POPF. The suture closure method may be a simple, safe, and effective method in dealing with BPD in PD.

## Introduction

Pancreaticoduodenectomy (PD) represents the standard surgical procedure for neoplasms of the pancreatic head and periampullary region. It involves the removal of the pancreatic head, duodenum, gallbladder, and common bile duct, with or without the removal of the gastric antrum; after resection, pancreaticojejunostomy (PJ), cholangiojejunostomy, and gastrojejunostomy must be performed. The most common complications after PD are delayed gastric emptying (DGE), pancreatic fistulae, hemorrhage, chyle leaks, endocrine and exocrine pancreatic insufficiency, and surgical site infections (1). The mortality rate of PD is about 2%–5%; the two most frequent causes of death were a leak from an anastomosis with sepsis/multiple organ system failure and bleeding. Clinically relevant postoperative pancreatic fistula (CR-POPF) occurs in up to 20% of patients and is typically associated with an increased hospital stay, cost, and reintervention rates. It is one of the most important initiating factors of severe complications and even death after PD (2).

The validated Fistula Risk Score (FRS) by Callery et al. (3) is the most cited and best used POPF prediction model. The FRS consists of gland texture, pancreatic duct diameter, intraoperative blood loss, and definitive pathology. The alternative Fistula Risk Score for PD (a-FRS) by Mungroop et al. (4) was based on pancreatic texture, duct diameter, and body mass index (BMI), without blood loss and pathology, and was successfully validated for both the 2005 and 2016 POPF definition. Kantor et al. (5) derived a modified Fistula Risk Score (mFRS) for preoperative risk stratification in patients undergoing PD, which included five predictors: sex, BMI, preoperative total bilirubin, pancreatic ductal diameter, and gland texture.

Except for the above risk factors, Shukla et al. (6) believed that the anatomy of the main pancreatic duct plays an important role in determining the outcomes of pancreatic anastomoses, and an investigation to identify its correlation is necessary. Bifid pancreatic duct (BPD) represents a relatively rare anatomical variation of the pancreatic ductal system, presenting a major bifurcation in the main pancreatic duct along its length. Halpert et al. (7) first reported a patient with bifid pancreas in 1990, diagnosed by ERCP. Steger et al. (8) investigated the anatomy of the pancreatic duct in 25 human cadaveric pancreas with a focus on the corpus area, and they found that in addition to the main and accessory pancreatic duct in the head, an additional BPD was observed within the pancreas corpus in 16% of the cases. Since then, there were some case reports about the BPD during PD (9–13). On 20 March 2015, we found two pancreatic duct orifices in the remnant of the pancreas during PD for the first time, and the BPD anatomy was confirmed *via* intraoperative probing, direct visualization of the ductal orifices, and dissecting the resected specimen postoperatively. As mentioned above, all the literature about the BPD during PD are case reports now; we do not know the incidence of BPD in PD, and the relationship between BPD and CR-POPF. This study aims to address these points.

## Patients and methods

### Patients and study design

**Figure 1:**
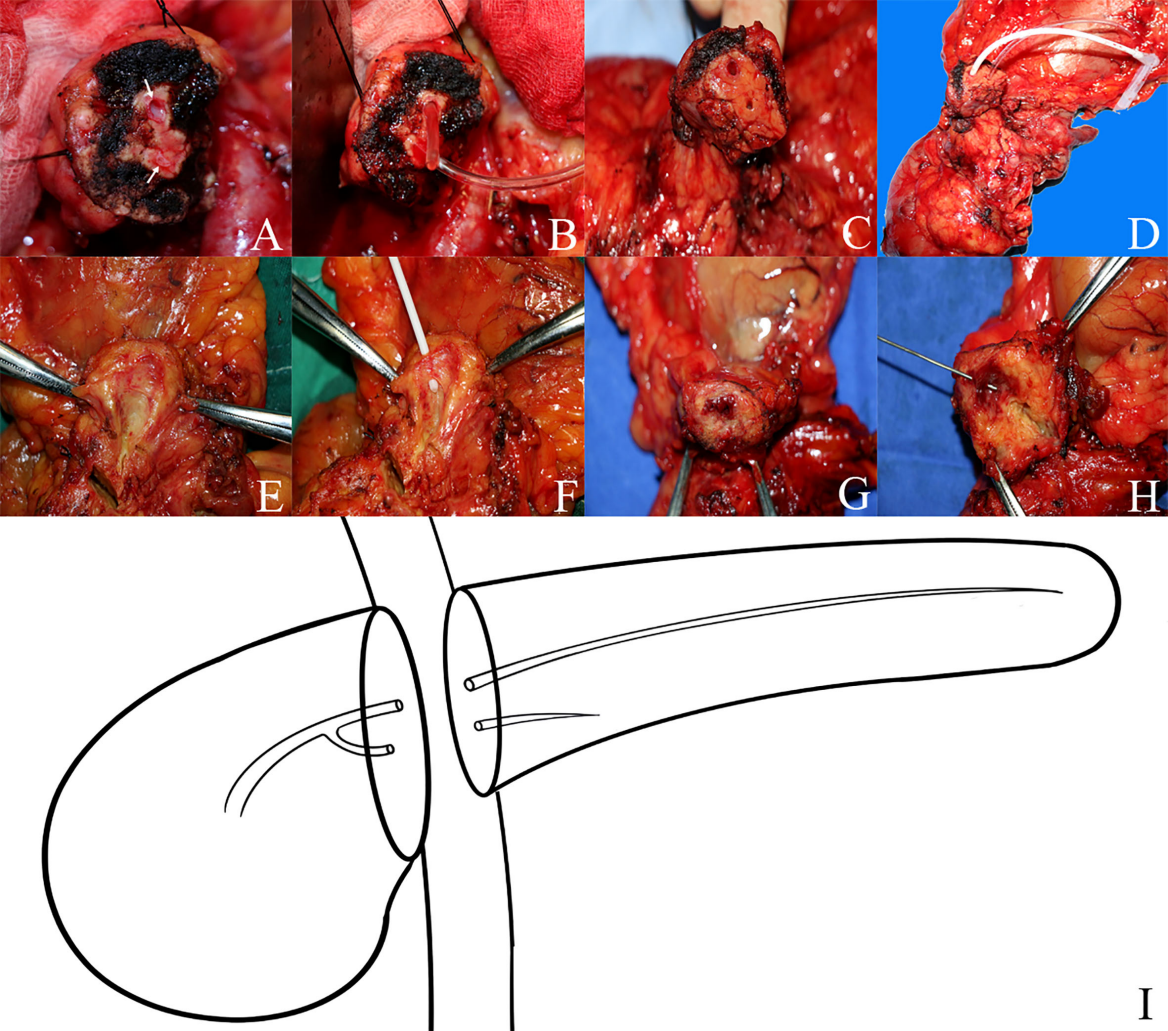
The anatomy of bifid pancreatic duct **(A–D)** Two pancreatic duct orifices in the remnant pancreas body during pancreaticoduodenectomy and the bifid pancreatic duct anatomy were confirmed *via* intraoperative probing and direct visualization of the ductal orifices. **(E–H)** The bifid pancreatic duct anatomy was confirmed by dissecting the resected specimen postoperatively; the bifid pancreatic duct in the body of the pancreas joins at the pancreatic head and drains through the major papilla. **(I)** Diagram of the anatomy of bifid pancreatic duct.

Between 20 March 2015 and 19 March 2016, 223 consecutive PDs were performed by a single surgeon in the Department of Hepatobiliary and Pancreatic Surgery (HBP Surgery) of Changhai Hospital Affiliated with the Naval Military Medical University. After transecting the pancreas, we inspected the cut surface of the residual dorsal pancreas carefully; if there are two pancreatic duct orifices in the remnant of the pancreatic body, and both of the two pancreatic ducts are more than 2 mm in diameter, we confirmed the BPD *via* intraoperative probing, direct visualization of the ductal orifices intraoperatively, and dissecting the resected specimen postoperatively (**Figure 1**).

In the former year (between 20 March 2014 and 19 March 2015), we did not pay attention to BPD, and all the 215 consecutive PDs performed by the same surgeon were treated as a single pancreatic duct (SPD). The preoperative imaging data of the two groups of patients were reviewed retrospectively, and all the patients were divided into four subgroups: imaging single pancreatic duct (I-SPD), imaging bifid pancreatic duct (I-BPD), operative single pancreatic duct (O-SPD), and operative bifid pancreatic duct (O-BPD). In I-BPD, the bifurcation and the joints of the two pancreatic ducts must be seen in the preoperative imaging.

### Pancreaticoduodenectomy and pancreatojejunostomy

The PD operation consists of an en bloc removal of the pancreatic head, the duodenum, the common bile duct, the gall bladder, and the distal portion of the stomach together with the adjacent lymph nodes. Pylorus-preserving pancreaticoduodenectomy (PPPD) leaves the functional pylorus at the gastric outlet. Because the neck of the pancreas is a vascular watershed between celiac and superior mesenteric arterial systems, the transection plane was 1.5–2.0 cm to the left of the neck of the pancreas (14, 15) in this study. In the O-BPD group, we sutured and ligated the small pancreatic duct with silk thread (suture closure method), and the large pancreatic duct was anastomosed with the jejunum by the double-layer continuous suturing PJ method (16) with 5-0 and 3-0 Prolene, respectively.

### Data collection and definition of postoperative complications

Demographic, histopathologic, and perioperative data of all the patients in the 2 years were collected comprehensively from the electronic medical record. The staging was based on the American Joint Committee on Cancer (AJCC) TNM Staging of Pancreatic Cancer (8th ed., 2017). The diagnosis of postoperative pancreatic fistulas (POPFs), postpancreatectomy hemorrhage (PPH), and delayed gastric emptying (DGE) was according to the International Study Group of Pancreatic Surgery (ISGPS) definition, POPF-ISGPS (2016) (17), PPH-ISGPS (2007) (18), and DGE-ISGPS (2007) (19), respectively. The assessment of postoperative complications was according to the Clavien–Dindo classification (2004) (20).

### Statistical method

Continuous variables were reported as median (interquartile range), and were compared using Mann–Whitney *U* test. Categorical variables were presented as whole numbers, and proportions and were compared by the *χ* ^2^ test or Fisher’s exact test when appropriate. The cutoff value of certain parameters was determined using the receiver operating characteristic curve. Logistic regression analyses were applied in univariate and multivariate risk factor analysis, then a nomogram was established for predicting CR-POPF according to the results of multivariate risk factor analysis. The nomogram was validated *via* concordance index analysis, receiver operating characteristic curve, and calibration plot. Statistical analyses were conducted using GraphPad Prism 9, SPSS 24.0 software, and R software version 4.1.2. All the statistical significance levels were two-sided, with *p*-values less than 0.05.

## Results

### Clinicopathological features

In this study, there were 438 consecutive PDs performed by the same surgeon, and they were divided into two groups: the former year group (between 20 March 2014 and 19 March 2015) and the latter year group (between 20 March 2015 and 19 March 2016). Of them, the former year group included 215 consecutive PDs, while the latter year group included 223. The main clinicopathological characteristics and postoperative complications of the two groups are shown in **Table 1**. There were no statistically significant differences in baseline characteristics among the two groups except that the postoperative complication is fewer in the latter year group compared with the former year group using the Clavien–Dindo classification (*p* = 0.036).

**Table 1:** Clinicopathological characteristics and postoperative complications.

Variable	Period		*p*-value	CR-POPF		*p*-value
	First year (*n* = 215)	Second year (*n* = 223)		Absent (*n* = 325)	Present (*n* = 113)	
Bifid pancreatic duct			0.178			0.032
Absent	206	207		311	102	
Present	9	16		14	11	
Gender			0.920			0.139
Female	82	83		129	36	
Male	133	140		196	77	
Age (years)			0.322			0.979
Male	60	52		83	29	
Female	155	171		242	84	
Smoking history			0.113			0.080
Absent	171	163		241	93	
Present	44	60		84	20	
History of alcoholism			0.293			0.405
Absent	188	202		287	103	
Present	27	21		38	10	
History of pancreatitis			0.525			0.609
Absent	195	207		297	105	
Present	20	16		28	8	
BMI			0.200			0.000
≤23.0	134	125		214	46	
≤23.0	81	97		111	67	
TNM stage			0.867			0.777
I-II	178	178		263	93	
III-IV	13	15		20	8	
Pathology			0.183			0.000
Other	102	113		137	188	
Pancreatic cancer and pancreatitis	121	102		86	27	
Postoperative blood transfusion		0.925			0.002
Absent	165	173		263	75	
Present	50	50		62	38	
High-grade antibiotic			0.327			0.000
Absent	140	156		287	9	
Present	75	67		38	104	
Intestinal fistula			NA			NA
Absent	215	223		325	113	
Present	0	0		0	0	
Chylous fistula			0.714			0.067
Absent	213	219		323	109	
Present	2	4		2	4	
Surgical site infection			0.058			0.000
Absent	134	159		288	5	
Present	81	64		37	108	
Pulmonary infection			0.466			0.000
Absent	209	220		324	105	
Present	6	3		1	8	
AP			1.000			0.005
Absent	211	219		323	107	
Present	4	4		2	6	
T-tube placement			0.423			0.238
Absent	204	216		309	111	
Present	11	7		16	2	
Clavien–Dindo Classification		0.036			0.000
1, 2	152	177		284	50	
3a, 3b, 4a, 4b, 5	63	46		41	63	
CR-POPF			0.227			NA
0	154	171				
1	61	52				
Blood type			0.573			0.813
O	73	67		107	33	
A	24	23		36	11	
B	57	56		81	32	
AB	61	77		101	37	
Postoperative abdominal hemorrhage		0.783			0.004
Absent	177	180		274	83	
Level B	33	39		48	24	
Level C	5	4		3	6	
Gastrointestinal hemorrhage		0.090			0.000
Absent	208	205		315	98	
Level B	5	14		8	11	
Level C	2	4		2	4	
DGE classification			0.399			0.003
0+A	182	195		289	88	
B+C	33	28		36	25	
NYHA score			0.102			0.173
1	117	125		182	60	
2	93	97		138	52	
3	5	0		5	0	
4	0	1		0	1	
ASA score			0.208			0.025
1	6	9		14	1	
2	164	181		261	84	
3	45	33		50	28	
NNIS score			0.597			0.279
0	126	140		195	71	
1	86	79		123	42	
2	3	4		7	0	
Rehospitalization			0.500			0.384
Absent	188	189		283	94	
Present	27	34		42	19	
Tumor diameter	3.08 ± 1.45	3.00 ± 1.43	0.568	3.17 ± 1.40	2.64 ± 1.50	0.001
Pancreatic duct diameter	0.50 ± 0.25	0.47 ± 0.27	0.156	0.52 ± 0.25	0.39 ± 0.26	0.000
Intraoperative bleeding	569.07 ± 419.04	487.22 ± 337.46	0.183	538.77 ± 403.23	494.69 ± 309.76	0.329
Total bilirubin	103.39 ± 117.86	95.99 ± 103.08	0.364	104.02 ± 108.07	86.9 ± 117.12	0.077
Direct bilirubin	75.72 ± 91.33	67.66 ± 79.4	0.806	74.42 ± 82.79	63.54 ± 92.88	0.178
Albumin	39.48 ± 3.04	39.98 ± 3.84	0.185	39.74 ± 3.56	39.7 ± 3.22	0.581
Alkaline phosphatase	279.98 ± 234.7	305.7 ± 289.1	0.777	594.31 ± 706.89	493.27 ± 735.88	0.107
Gamma-glutamyl transpeptidase	512.64 ± 579.8	624.34 ± 824.24	0.676	12.7 ± 0.97	12.48 ± 0.84	0.088
Hemoglobin	126.02 ± 16.41	125.5 ± 16.3	0.542	124.74 ± 15.37	128.69 ± 18.6	0.005
Platelet	235.56 ± 84.24	232.94 ± 88.06	0.843	230.91 ± 87.44	243.74 ± 81.8	0.056
C-reactive protein	17.52 ± 23.52	13.99 ± 19.32	0.669	14.37 ± 20.65	21.47 ± 25.13	0.295
CA199	163.83 ± 239.78	148.68 ± 215.16	0.615	174.94 ± 236.18	101.8 ± 189.09	0.001
Carcinoembryonic antigen	4.99 ± 5.62	7.86 ± 24.04	0.627	6.54 ± 16.45	6.36 ± 21.81	0.068
Alpha fetoprotein	23.59 ± 167.85	24.87 ± 183.51	0.598	18.35 ± 153.01	41.37 ± 229.96	0.047
CA153	14.89 ± 24.41	11.12 ± 5.07	0.441	13.55 ± 19.42	10.76 ± 4.74	0.198
CA724	3.23 ± 4.31	5.62 ± 21.61	0.160	5.12 ± 19.04	3 ± 2.97	0.736
Hospitalization days	13.93 ± 10.27	12.18 ± 8.88	0.661	10.43 ± 5.77	20.57 ± 13.72	0.000

p < 0.05 by Continuity Correction χ^2^ test for count data and p < 0.05 by Mann–Whitney U test for continuous data.

CR-POPF, Clinically relevant postoperative pancreatic fistula.

PD, Pancreaticoduodenectomy.

PPPD, Pylorus-preserving pancreaticoduodenectomy.

PV/SMV, Portal Vein/Superior Mesenteric Vein.

NYHA, New York Heart Association classification.

ASA, American Society of Anesthesiologists.

APACHE, Acute Physiology And Chronic Health Evaluation scoring system.

NNIS, National Nosocomial Infections Surveillance risk index.

DGE, Delayed gastric emptying.

### The incidence of bifid pancreatic duct in pancreaticoduodenectomy

**Figure 2:**
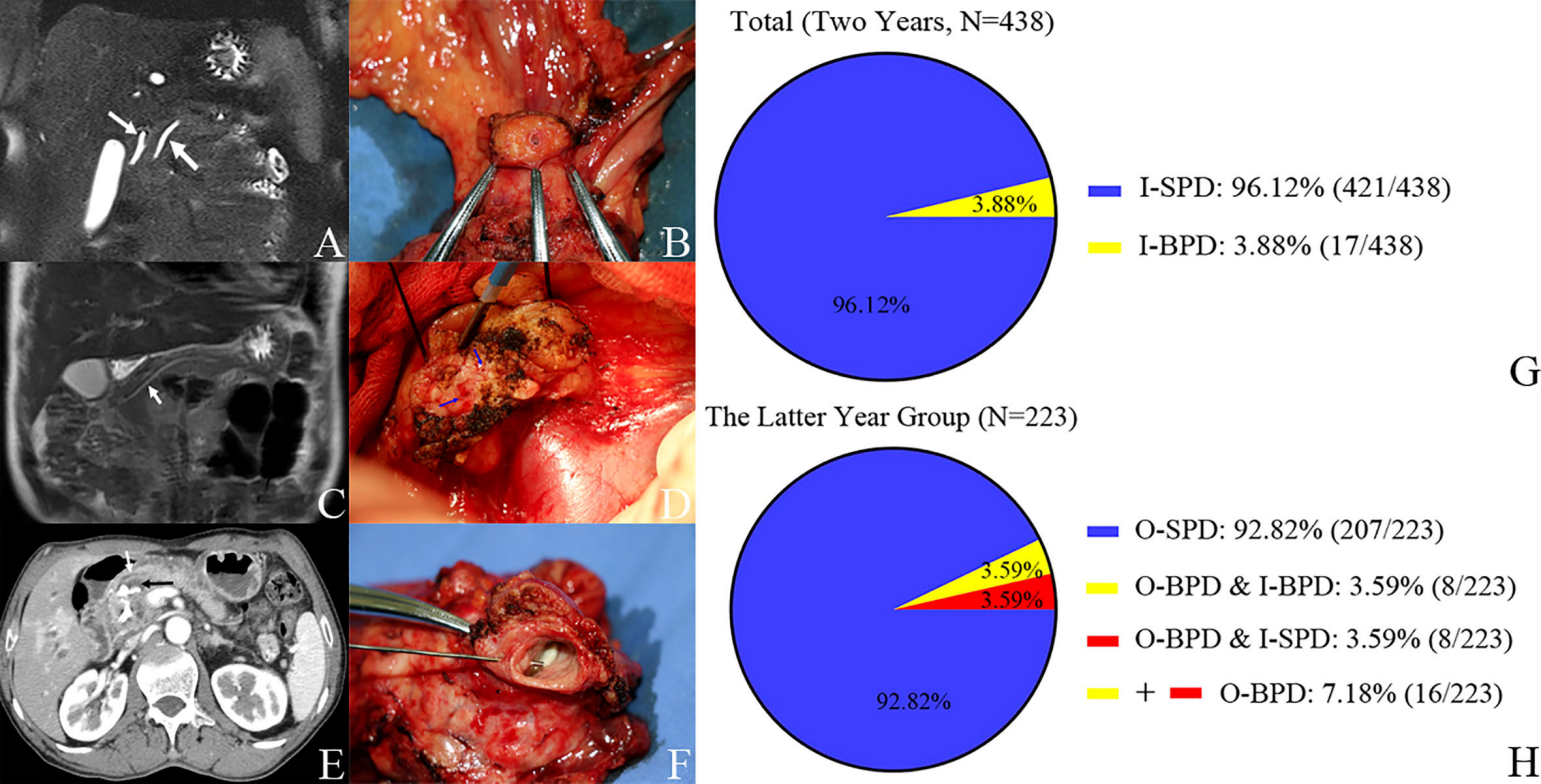
Pancreatic duct in preoperative imaging and operation and the incidence of bifid pancreatic duct. Case 1. **(A)** I-SPD vs. **(B)** O-SPD. **(A)** I-SPD: common bile duct (left arrow) and main pancreatic duct (right arrow) in preoperative imaging. **(B)** O-SPD: one pancreatic duct orifice in the remnant of pancreas. Case 2. **(C)** I-SPD vs. **(D)** O-BPD. **(C)** I-SPD: main pancreatic duct (arrow) in preoperative imaging. **(D)** O-BPD: bifid pancreatic duct (two green arrows) in pancreaticoduodenectomy. Case 3. **(E)** I-BPD vs. **(F)** O-BPD. **(E)** I-BPD: bifid pancreatic duct (white arrow and black arrow) in preoperative imaging. **(F)** O-BPD: bifid pancreatic duct in resected specimen; the bifid pancreatic duct in the body of the pancreas joins at the pancreatic head (probe in the small pancreatic duct). **(G, H)** Incidence of bifid pancreatic duct in preoperative imaging and operation. I-SPD, imaging single pancreatic duct; O-SPD, operative single pancreatic duct; I-BPD, imaging bifid pancreatic duct; O-BPD, operative bifid pancreatic duct.

In the latter year group, we found 16 BPDs during PD (O-BPD); thus, the incidence of O-BPD is 7.18% (16/223). Of them, there were eight patients who had BPD in the preoperative imaging (I-BPD). All the I-BPDs are O-BPDs; it means that 50% of O-BPDs were SPD in the preoperative imaging (I-SPD). There were 17 BPDs in the 438 consecutive PDs in the preoperative imaging (I-BPD); thus, the incidence of I-BPD is 3.88% (17/438) in this study (**Figure 2**).

### The effect of bifid pancreatic duct on postoperative complications in PD

There were no statistically significant differences in preoperative baseline characteristics among the two groups, including the rate of CR-POPF (*p* = 0.227). In the former year group, the rate of severe complications (Clavien–Dindo classification ≥ IIIa) of I-BPD and I-SPD is 77.78% (7/9) and 27.18% (56/206), respectively (*p* = 0.003); the rate of CR-POPF of I-BPD is higher than I-SPD, 55.56% (5/9) vs. 27.18% (56/206), but there were no statistically significant differences (*p* = 0.122). In the latter year group, the rate of severe complications of O-BPD and O-SPD is 50% (8/16) and 18.36% (38/207), and the rate of CR-POPF of O-BPD and O-SPD is 37.5% (6/16) and 22.22% (46/207), respectively; both of them have statistically significant differences, and the *p*-value is 0.003 and 0.006, respectively. In the subgroup analysis, both the rate of severe complications and the rate of CR-POPF of I-BPD were higher than O-BPD, 77.78% (7/9) vs. 50% (8/16), and 55.56% (5/9) vs. 37.5% (6/16), but there were no statistically significant differences in both of them; the *p*-value is 0.174 and 0.434, respectively. The effect of BPD on postoperative complications in PD is shown in **Table 2**.

**Table 2:** Bifid pancreatic duct and postoperative complications.

Variable	Severe complications		*p*-value	CR-POPF		*p*-value
	Absent	Present		Absent	Present	
Bifid pancreatic duct			<0.001			0.032
Absent (*n* = 413)	319	94		311	102	
Present (*n* = 25)	10	15		14	11	
The former year I-BPD			0.003			0.122
Absent (*n* = 206)	150	56		150	56	
Present (*n* = 9)	2	7		4	5	
The latter year O-BPD			0.003			0.006
Absent (*n* = 207)	169	38		161	46	
Present (*n* = 16)	8	8		10	6	
I-BPD and O-BPD			0.174			0.434
The former year I-BPD (*n* = 9)	2	7		4	5	
The latter year O-BPD (*n* = 16)	8	8		10	6	

p < 0.05 by Continuity Correction χ^2^ test for count data.

Severe complications: complications of level 3, level 4, and level 5 according to Clavien–Dindo classification.

### Univariate and multivariate analyses of the factors associated with CR-POPF

The perioperatively obtained variables, include age, gender, hypertension, cardiovascular disease, smoking history, history of alcoholism, history of pancreatitis, history of abdominal surgery, BMI, TNM stage, pathology, blood type, NYHA score, ASA score, NNIS score, APACHE score, intraoperative bleeding, intraoperative blood transfusion, and indexes of blood or serum tests, were subjected to univariate and multivariate analyses. Our results showed that BPD (hazard ratio [HR] 2.396, 95% confidence interval [CI] 1.054–5.433), pancreatic duct diameter <0.2 cm (3.515, 2.041–6.054), tumor diameter ≤2 cm (3.31, 2.021–5.423), ASA score (1.914, 1.186–3.089), pathology except for pancreatic cancer and pancreatitis (4.371, 2.691–7.101), and BMI ≥ 23 (2.808, 1.809–4.359) were independent risk factors of CR-POPF (**Table 3**).

**Table 3:** Univariate and multivariate factor analysis.

Variable	Univariate				Multivariate			
	*p*	HR	Lower limit	Upper limit	*p*	HR	Lower limit	Upper limit
Bifid pancreatic duct, present: absent	0.037	2.396	1.054	5.443	0.000	7.115	2.590	19.548
Pancreatic duct diameter, <0.2:≧0.2	0.000	3.515	2.041	6.054	0.013	2.328	1.192	4.548
Tumor diameter, ≤2:>2	0.000	3.310	2.021	5.423	0.000	3.090	1.706	5.597
ASA score, 3:2:1	0.008	1.914	1.186	3.089	0.000	3.339	1.827	6.103
Pathology, other: Pancreatic cancer and pancreatitis	0.000	4.371	2.691	7.101	0.000	3.522	1.926	6.440
BMI, ≥23:<23	0.000	2.808	1.809	4.359	0.000	2.834	1.648	4.875

p < 0.05 by Logistic regression model.

Cutoff value of tumor diameter was calculated by ROC curve.

### Construction and validation of nomogram

**Figure 3:**
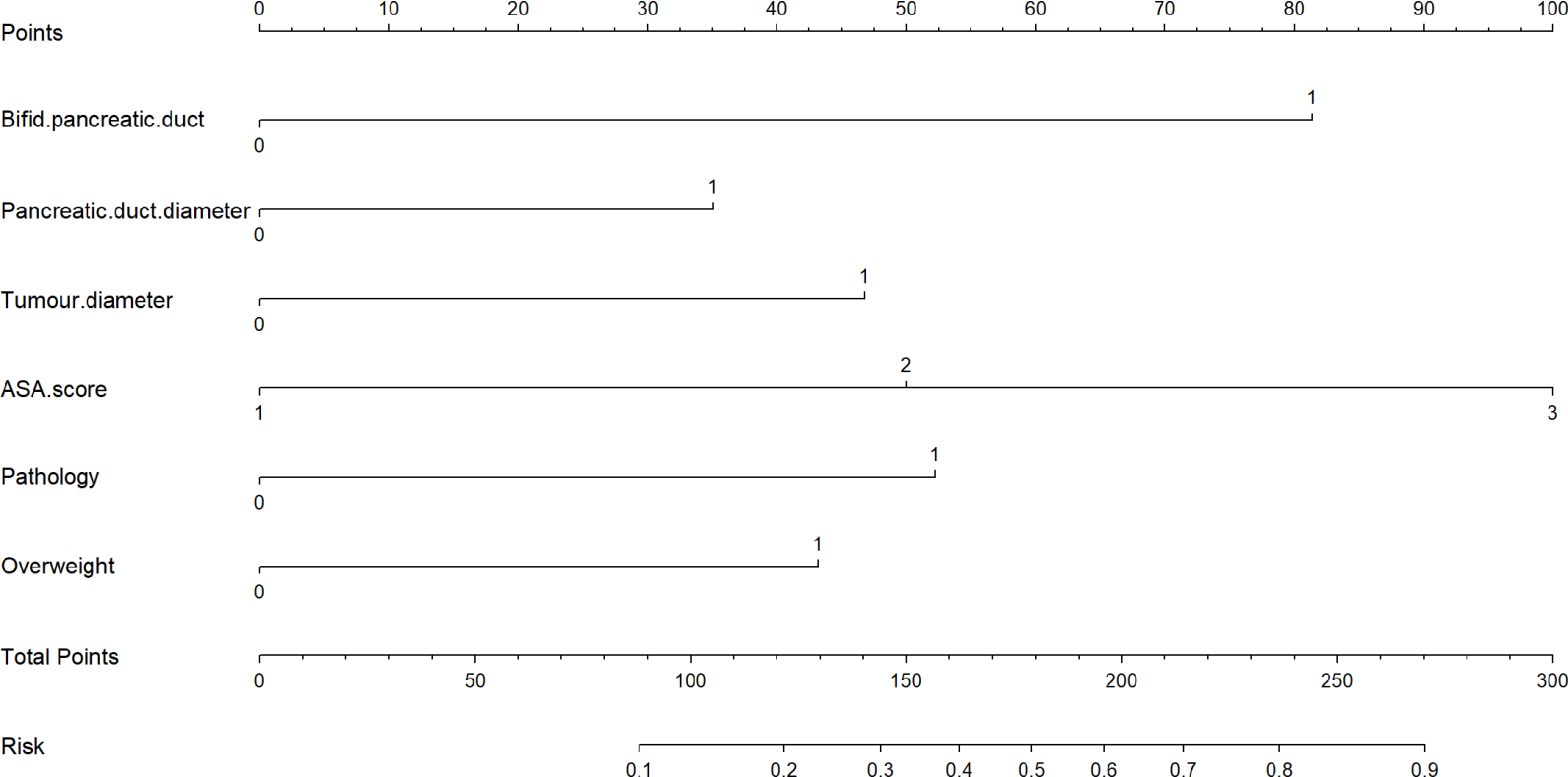
** **A novel nomogram for predicting CR-POPF of patients who underwent pancreaticoduodenectomy. The nomogram is used by adding up the points identified on the points scale for each variable. According to the sum of these points projected on the bottom scales, the nomogram can provide the incidence of CR-POPF for an individual patient. CR-POPF, clinically relevant postoperative pancreatic fistula.

**Figure 4:**
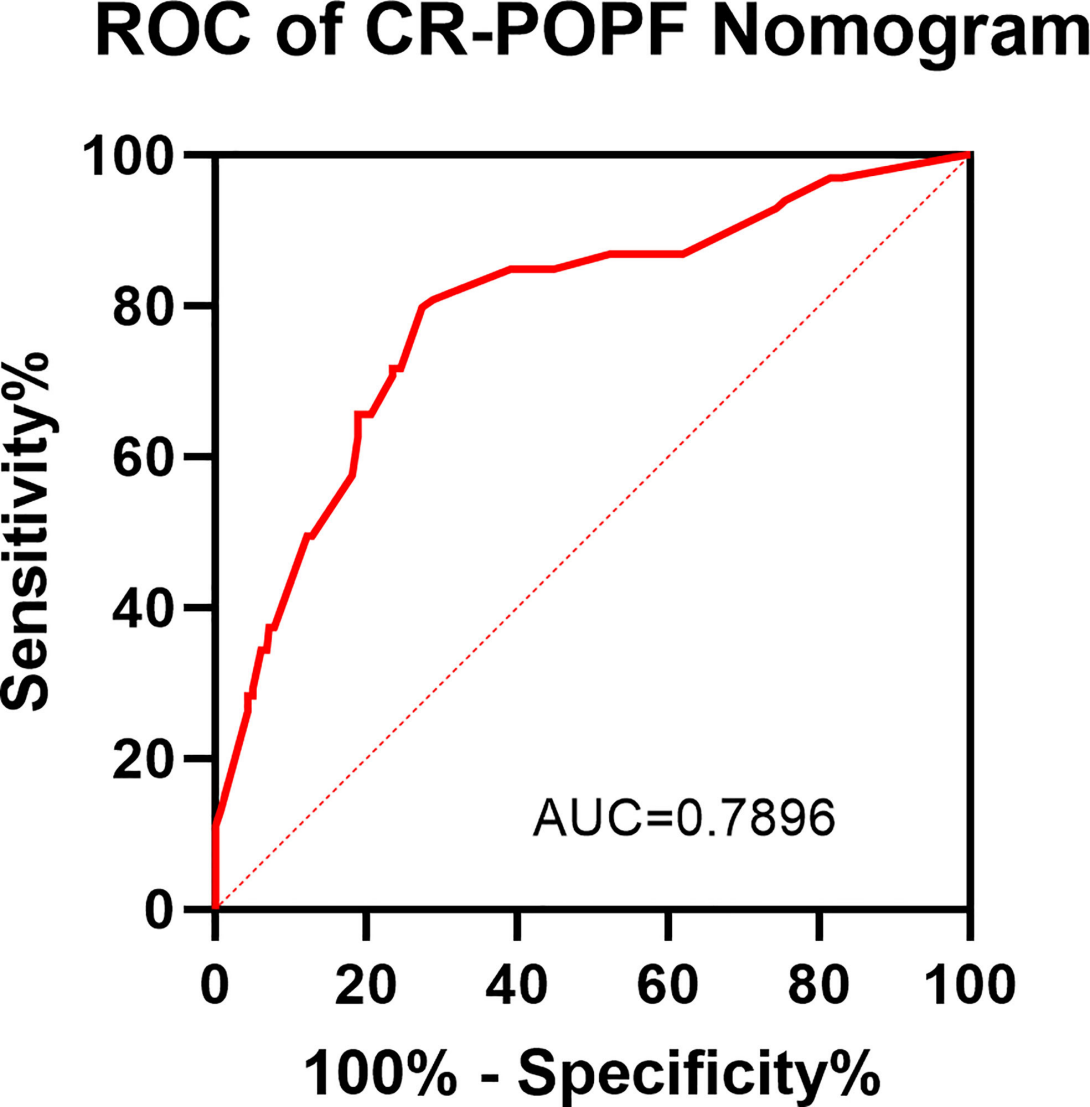
ROC of the CR-POPF nomogram. A receiver operating characteristic (ROC) curve was conducted for assessing the model. The area under the ROC curve is 0.7896.

**Figure 5:**
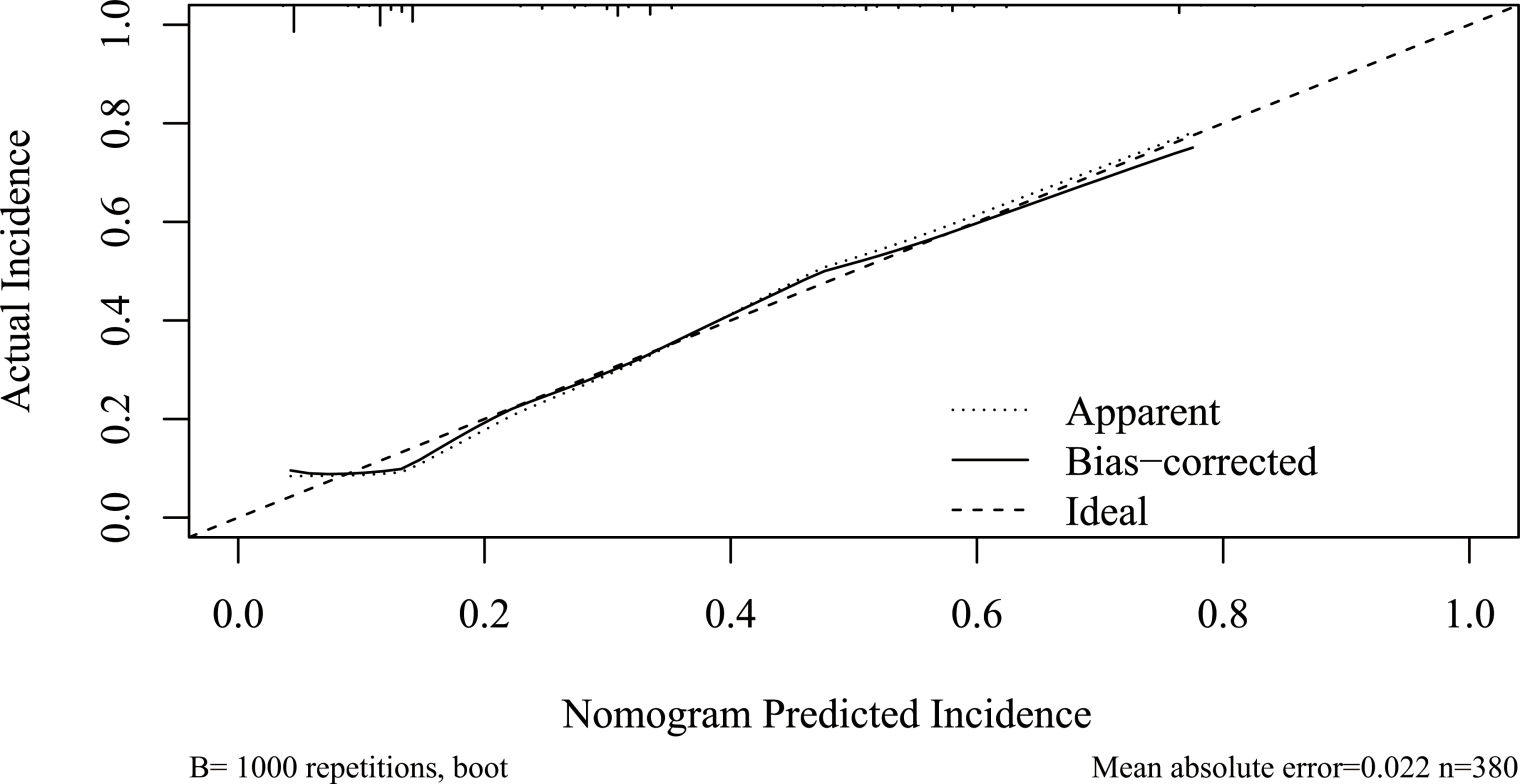
Calibration plot of the nomogram. Calibration plot of the nomogram predicting CR-POPF. The *x*-axis represents the nomogram-predicted survival, and the actual survival is plotted on the *y*-axis. The apparent incidence of CR-POPF, the ideal incidence, and the bias-corrected incidence were shown as different lines. CR-POPF, clinically relevant postoperative pancreatic fistula.

As listed in **Table 3**, BPD, pancreatic duct diameter <0.2 cm, tumor diameter ≤2 cm, ASA score, pathology except for pancreatic cancer and pancreatitis, and BMI ≥ 23 were selected in the construction of nomogram predicting CR-POPF (**Figure 3**). The concordance index was 0.795, and the area under the curve (AUC) was 0.790 according to the receiver operating characteristic (ROC) curve (**Figure 4**). The concordance index is a measure of the predictive accuracy of the model being tested, which ranges from 0.5 (completely random prediction) to 1 (perfect prediction). The apparent incidence of CR-POPF, the ideal incidence, and the bias-corrected incidence were shown as different lines in a calibration plot (**Figure 5**). The bias-corrected (also known as overfitting-corrected or optimism-corrected) line is produced using a bootstrap approach to estimate predicted and observed values based on a nonparametric smoother applied to a sequence of predicted values. These three lines were closely aligned, demonstrating good calibration.

## Discussion

**Figure 6:**
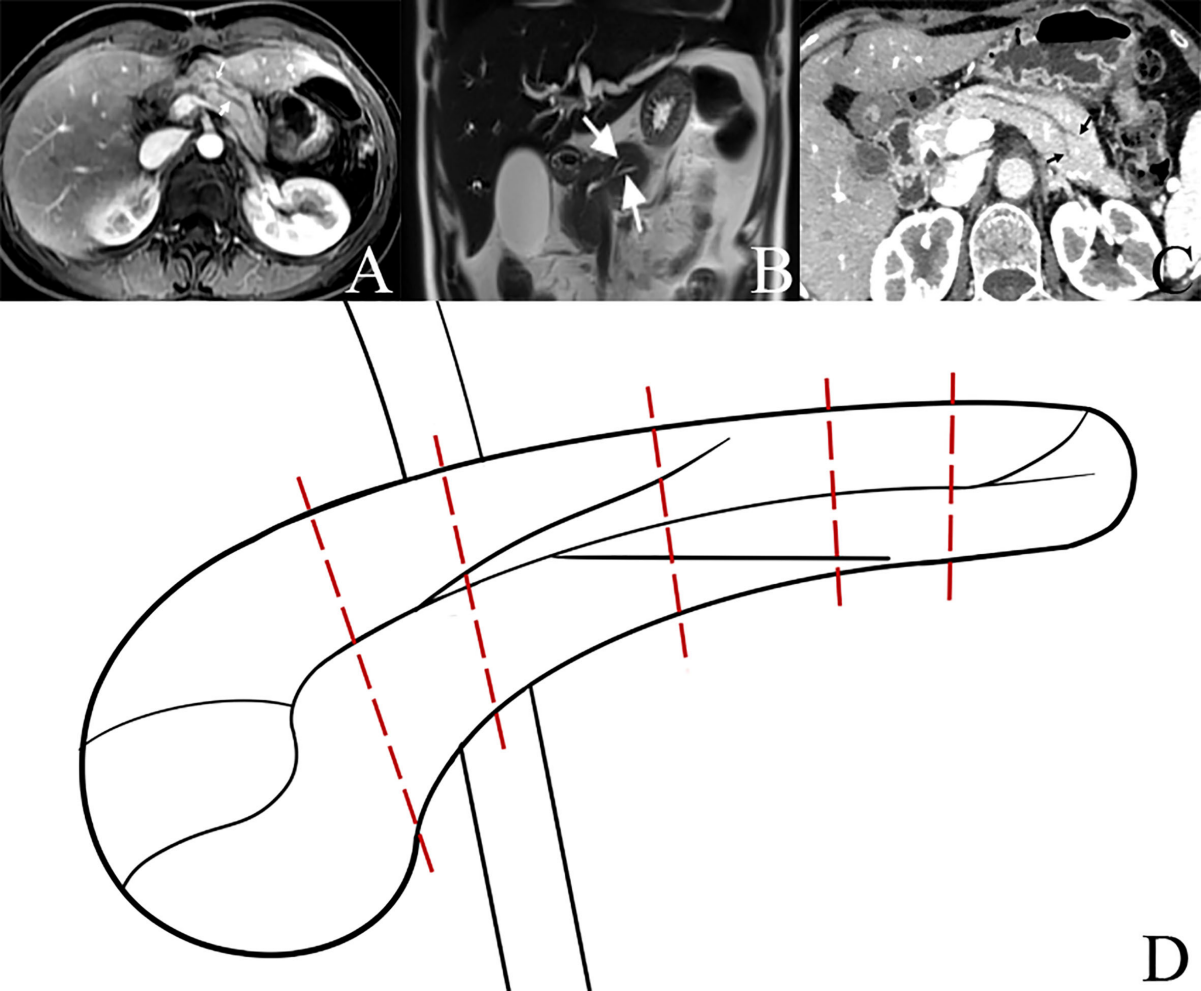
Bifid pancreatic duct at the neck, body, and tail of pancreas. **(A)** Bifid pancreatic duct (two white arrows) at the neck of pancreas. **(B)** Bifid pancreatic duct (two white arrows) at the body of pancreas. **(C)** Bifid pancreatic duct (two black arrows) at the tail of pancreas. **(D)** Diagram of bifid pancreatic duct at different locations of pancreas; different pancreatic transections result in different numbers of pancreatic duct orifices in the remnant of pancreas during operation.

On 20 March 2015, we found BPD in the remnant pancreatic body during PD for the first time, and the BPD anatomy was confirmed *via* intraoperative probing, direct visualization of the ductal orifices, and dissecting of the resected specimen postoperatively. BPD represents a relatively rare anatomical variation of the pancreatic ductal system, presenting a major bifurcation in the main pancreatic duct along its length; it is different from the main pancreatic duct (Wirsung duct) and accessory pancreatic duct (Santorini duct) in the head of the pancreas (**Figure 1**). Steger et al. (8) investigated the anatomy of the pancreatic duct in 25 human cadaveric pancreas with a focus on the corpus area, and they found BPD within the pancreas corpus in 16% of the cases (**Figure 6**). There are also some case reports about the BPD during PD (9–13), and since all the literature about BPD are published as case reports, the incidence of BPD in PD remains unclear. In this study, we defined BPD as the diameter of both pancreatic ducts larger than 2 mm.

In this study, during PD, the incidence of O-BPD is 7.17% (16/223), and 50% of O-BPDs were SPD in the preoperative imaging (I-SPD); thus, during PD, a careful intraoperative inspection of the cut surface of the residual dorsal pancreas is needed to identify the presence of BPD even in I-SPD patients. There were 17 BPDs in the 438 consecutive PDs in the preoperative imaging (I-BPD); thus, the incidence of I-BPD is 3.88% (17/438). Obstruction of the pancreatic duct and transecting the pancreas at the left of the pancreatic neck may increase the incidence of BPD in PD (8).

**Figure 7:**
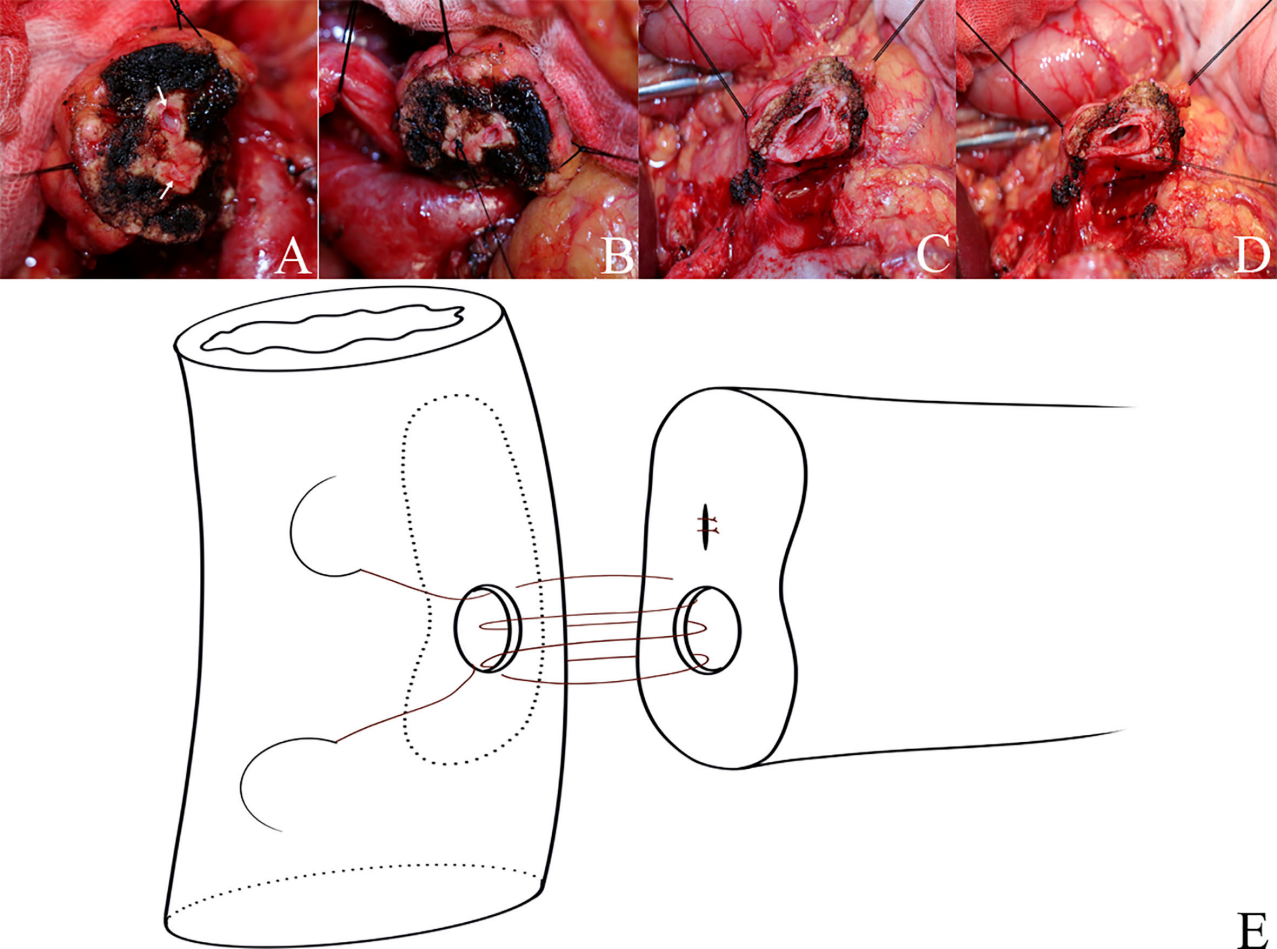
Suture closure method and double-layer continuous suturing pancreaticojejunostomy. **(A–D)** Suture closure method (suture and ligate the small pancreatic duct with silk thread). **(E)** The large pancreatic duct was anastomosed with the small intestine by double-layer continuous suturing pancreaticojejunostomy with 5-0 and 3-0 Prolene, respectively.

There are two pancreatic duct orifices in the remnant pancreatic body in patients with BPD during PD; in this study, we found that both the rate of severe complications and the rate of CR-POPF of BPD were higher than SPD, and both the rate of severe complications and the rate of CR-POPF of I-BPD in the former year (untreated) were higher than O-BPD (treated with the suture closure method), suggesting that we must deal with the BPD in PD. Yoshida et al. (9) and Vasiliadis et al. (10) reported one case of double duct-to-mucosa PJ for BPD following PD. On the other hand, Ball et al. (11) and Shim et al. (12) sutured and ligated the small BPD, and the large pancreatic duct was anastomosed with the jejunum using a standard duct-to-mucosa PJ. Recently, Ishida et al. (13) presented a novel technique named the “two-in-one” method in a case of PD for BPD; they anastomosed one jejunal hole to a double pancreatic duct. In this study, we sutured and ligated the small pancreatic duct with silk thread (suture closure method) in O-BPD patients, and the large pancreatic duct was anastomosed with the jejunum by double-layer continuous suturing PJ (16) with 5-0 and 3-0 Prolene, respectively (**Figure 7**). By using the suture closure method, both the rate of severe complications and the rate of CR-POPF decreased, suggesting that the suture closure method may be a simple, safe, and effective method in dealing with BPD in PD. Different from duct-to-mucosa PJ, invagination PJ is performed by invagination of 1–2 cm of the proximal end of the pancreatic stump into the jejunum (21); thus, invagination PJ may be superior to duct-to-mucosa PJ in patients with BPD.

In the study of the anatomy of the pancreatic duct in the human cadaveric pancreas, fewer transversely cut side branches were observed within the plane of the portal vein as compared to the resection planes 2–4 cm beneath (8), suggesting that it is appropriate to transect the pancreas within the plane of the portal vein when it does not affect the radical PD.

When the transection plane of the pancreas is obscured by burning or bleeding during PD, intraoperative ultrasonography (IOUS) was useful in identifying the exact location of the BPD. Ohkubo et al. (22) used the IOUS and IOUS-guided pancreatography to clarify the exact location of BPD during PD; the transection line was set on the proximal side of the pancreatic duct bifurcation, which helped prevent the inadvertent suture of the second pancreatic duct or leave the second duct without anastomosis, which may result in CR-POPF and severe complications. By using IOUS to confirm the exact location of the pancreatic duct bifurcation as well as the tumor extension, Tajima et al. (23) performed a distal pancreatectomy, instead of a middle pancreatectomy, with a cutting line at the downstream pancreas to the duct bifurcation point, which resulted in a favorable outcome without any postoperative complications. It suggests that IOUS-guided pancreatography should be recommended to confirm the relationship between the transection line of the pancreas and the duct bifurcation point when performing PD if BPD is suspected.

Postoperative pancreatic fistula (POPF) is one of the most threatening complications after PD. POPF occurs in up to 20% of patients and is typically associated with an increased hospital stay, cost, reintervention rates, and mortality. Different factors may predict POPF, including gland texture, pancreatic duct diameter, intraoperative blood loss, definitive pathology, BMI, sex, and preoperative total bilirubin (3–5). Except for the above risk factors, the anatomy of the main pancreatic duct plays an important role in determining the outcomes of pancreatic anastomoses (6). BPD represents a relatively rare anatomical variation of the pancreatic ductal system, presenting a major bifurcation in the main pancreatic duct along its length. Our results showed that BPD, pancreatic duct diameter <0.2 cm, tumor diameter ≤2 cm, ASA score, pathology except for pancreatic cancer and pancreatitis, and BMI ≥ 23 were independent risk factors of CR-POPF.

Nonetheless, our study has some limitations. First, this study is a single-center retrospective study; a selection bias may be suggested by the retrospective nature. Second, BPD in the former year group was not intraoperatively investigated and estimation of I-BPD is difficult even after reviewing MR imaging, and as the incidence of BPD is low, and the sample size of this study was not large enough, we put the I-BPD cases from the former year group together with the O-BPD from the latter year group in the univariate and multivariate analyses. Third, as mentioned above, because the incidence of BPD is low, and the sample size of this study was not large enough, we have not performed a propensity matching score (PSM) study. Fourth, BPD should be treated during PJ; in this article, because we only used the suture closure method, we do not know the result of other methods [such as double duct-to-mucosa PJ (9, 10) and the two-in-one method (13)]; hence, a large-sample-size, multicenter, and randomized controlled trial needs to be performed in the future.

In conclusion, in this study, the incidence of O-BPD is 7.18% during PD, 50% of O-BPDs were SPD in the preoperative imaging (I-SPD), and the incidence of I-BPD is 3.88%. BPD is an independent risk factor of CR-POPF after PD, and the suture closure method may be a simple and safe method in dealing with BPD, with a potential reduction of CR-POPF rate, although the effectiveness still needs to be proven in further clinical research.

## Data availability statement

The original contributions presented in the study are included in the article/supplementary material. Further inquiries can be directed to the corresponding authors.

## Ethics statement

This study was reviewed and approved by the Ethics Committee of Changhai hospital. The patients/participants provided their written informed consent to participate in this study.

## Author contributions

Contributions: (I)†contribute equally; (II)Conception and design: GJ, X-GH, and GL. (III) Administrative support: GJ, X-GH, and GL. (IV) Provision of study patients: GJ, LO, ZS, K-LZ, WJ. (V) Collection and assembly of data: LO, HH, GN. (VI) Data analysis and interpretation: All authors; (VII) Manuscript writing: LO. All authors contributed to the article and approved the submitted version.

## Funding

This study was supported by the Shanghai ShenKang hospital development center (No. SHDC2020CR2001A).

## Conflict of interest

The authors declare that the research was conducted in the absence of any commercial or financial relationships that could be construed as a potential conflict of interest.

## Publisher’s note

All claims expressed in this article are solely those of the authors and do not necessarily represent those of their affiliated organizations, or those of the publisher, the editors and the reviewers. Any product that may be evaluated in this article, or claim that may be made by its manufacturer, is not guaranteed or endorsed by the publisher.
